# Fabrication of Wide–Range–Visible Photocatalyst Bi_2_WO_6−x_ nanoplates via Surface Oxygen Vacancies

**DOI:** 10.1038/srep19347

**Published:** 2016-01-18

**Authors:** Yanhui Lv, Wenqing Yao, Ruilong Zong, Yongfa Zhu

**Affiliations:** 1 Department of Chemistry, Beijing Key Laboratory for Analytical Methods and Instrumentation, Tsinghua University, Beijing, 100084, People’s Republic of China; 2 Key Laboratory of Photochemistry Beijing National Laboratory for Molecular Sciences Institute of Chemistry, Chinese Academy of Sciences, Beijing 100190, People’s Republic of China

## Abstract

Bi_2_WO_6_ as a high visible-light-driven catalyst has been aroused broad interest. However, it can only be excitated by the light with λ < 450 nm and the solar energy utilization need to be improved. Here, the wide–range–visible photoresponse Bi_2_WO_6−x_ nanoplates were fabricated by introducing surface oxygen vacancies through the controllable hydrogen reduction method. The visible photoresponse wavelength range is extended from 450 nm to more than 600 nm. In addition, the photocatalytic activity of Bi_2_WO_6−x_ is also increased and is 2.1 times as high as that of pristine Bi_2_WO_6_. The extending of the photoresponse range and the enhancement of the photoactivity both can be attributed to the surface-oxygen-vacancy states. This is because surface-oxygen–vacancy states generated above and partly overlapping of with the valence band (VB) will result in the rising of valence band maximum (VBM), thus broadening the VB width. This approach is proposed to develop many types of wide–range–visible optical materials and to be applicable to many narrow and wide bandgap materials.

For decades, photocatalysis as a green chemistry technology using sunlight has been attracting tremendous attention. Photocatalysis can completely decompose organic pollutants even at low levels in an ambient environment. Bi_2_WO_6_ has attracted considerable attention for its physical and chemical properties, such as pyroelectricity, ferroelectricity, piezoelectricity and non–linear dielectric susceptibility^1^,^2^,^3^,^4^,^5^,^6^,^7^. Besides, Bi_2_WO_6_ is also a kind of excellent visible–light–driven photocatalysts on the degradation of organic pollutants and water splitting (O_2_ evolution)^8^,^9^,^10^,^11^,^12^,^13^,^14^. The high photoactivity of Bi_2_WO_6_ could be attributed to the corner–sharing structure of WO_6_ octahedron sandwiched between (Bi_2_O_2_)^2+^ layers, which is conducive to the transfer of electrons to the surface of the photocatalyst along the layered network because the recombination of photogenerated electron and hole pairs can be suppressed by the electron transferring to a layered host^11^,^12^,^15^. Kudo and Hijii first reported the photocatalytic O_2_ evolution by Bi_2_WO_6_ from AgNO_3_ solution^11^. Zou and Ye *et al*. demonstrated that Bi_2_WO_6_ possessed the photocatalytic activity for O_2_ evolution, as well as the activity of mineralizing both CHCl_3_ and CH_3_CHO to CO_2_ under visible–light irradiation^12^. Furthermore, in order to increase the utilization efficiency of sunlight, many works have been researched on the enhanced of photoactivity, such as F–Bi_2_WO_6_ 10, Co_3_O_4_–Bi_2_WO_6_ 16, AgBr–Ag–Bi_2_WO_6_ 17, Er^3+^–Bi_2_WO_6_ 18, Gd–Bi_2_WO_6_ 19, C_60_–Bi_2_WO_6_ 20, C–Bi_2_WO_6_ 21, Ag–Bi_2_WO_6_ 22, Ce and F codoped Bi_2_WO_6_ 23 etc. However, these composite photocatalysts either contain toxic ions (Co^3+^) or are expensive (Er^3+^, C_60_, Ag) or possess complex synthetic process. Therefore, it is still a great challenge to find a facile, economical, environmentally benign method to fabricate high–efficient Bi_2_WO_6_–based photocatalysts. It is well known that the defect structure is one of the important factors on the photocatalytic performance. Recently, some works have been founded that the amazing generation of the visible photoactivity for the UV lighted photocatalyst (mainly TiO_2_ and ZnO) by introducing oxygen vacancies^24^,^25^,^26^,^27^. In our previous work, simple semiconductor ZnO and high UV photoactivity BiPO_4_ photocatalysts with surface oxygen vacancies were successfully fabricated via two facile, economical and highly efficient methods, vacuum deoxidation and controllable hydrogen reduction^28^,^29^,^30^. After surface oxygen vacancies were introduced in the photocatalyst, the photoresponse wavelength range is extended as well as the UV photoactivity is greatly improved.

In this work, Bi_2_WO_6−x_ photocatalyst with wide–range–visible response and high–visible activity was fabricated by introducing surface oxygen vacancies. The formation process of oxygen vacancies has been discussed in detail, and the influences of the oxygen vacancy extent on the photoabsorption properties, electric potential and electronic structure also have been investigated. The roundly mechanism of the increased photocatalytic efficiency and activity has been provided. Furthermore, this work is a supplement for illustrating that it is a universal way that can extend the photoresponse range as well as enhance photoactivity by introducing surface oxygen vacancies.

## Results and Discussions

### Enhancement of Photocatalytic Activity

**Figure 1:**
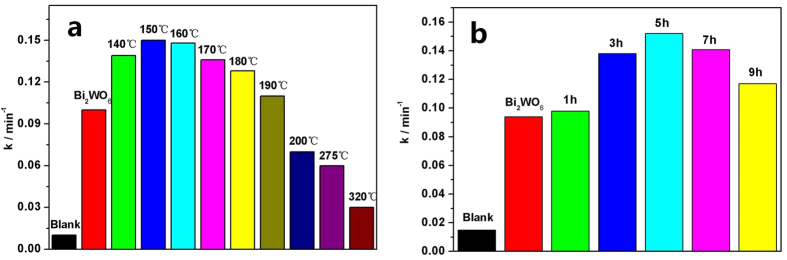
Visible Photocatalytic activity of pristine Bi_2_WO_6_ and Bi_2_WO_6−x_ after hydrogen reduction (**a**) at various temperature for 5 h; (**b**) at 150 °C for various time on the degradation of 2, 4–DCP, λ > 420 nm.

Visible photocatalytic activities of Bi_2_WO_6_ and Bi_2_WO_6−x_ samples after hydrogen treated with various temperature for 5 h and various time at 150 °C on the degradation of 2, 4–dichlorophenol (2, 4–DCP), under λ > 420 nm light, are shown in Fig. 1a,b. The degradation process is fitted to pseudo–first–order kinetics, and the value of the rate constant *k* is equal to the corresponding slope of the fitting line. The visible photocatalytic activities of Bi_2_WO_6−x_ samples gradually enhanced with the increase of hydrogen reduction temperature and time; when the temperature reaches 150 °C, time is for 5 h, Bi_2_WO_6−x_ displays the highest photodegradation activity. The apparent rate constant *k* is 0.152 min^−1^ and it is about 1.6 times as high as that of pristine Bi_2_WO_6_ (*k* = 0.093 min^−1^). However, further increasing the temperature or prolonging the time, the degradation rate begins to decrease. Even when the temperature is higher than 200 °C (such as at 275, 320 °C), the photoactivity of Bi_2_WO_6−x_ is lower than that of pristine Bi_2_WO_6_. The photocatalytic performance of Bi_2_WO_6−x_ photocatalysts are greatly influenced by the number and kind of oxygen vacancies and the reduction degree of Bi_2_WO_6_ (for example after 320 °C, 5 h hydrogen reduction, metal Bi is generated), which are controlled by tuning the temperature and time in the process of hydrogen reduction. As is well known, high concentration of surface oxygen vacancies contribute to the separation efficiency of photogenerated electron–hole pairs, improving the photocatalytic activity; while bulk oxygen vacancies as charge capture center will inhibit the photoactivity^31^. In addition, metal Bi has no photocatalytic activity, and its existence is infaust for the photocatalytic performance of Bi_2_WO_6_ 32.

**Figure 2:**
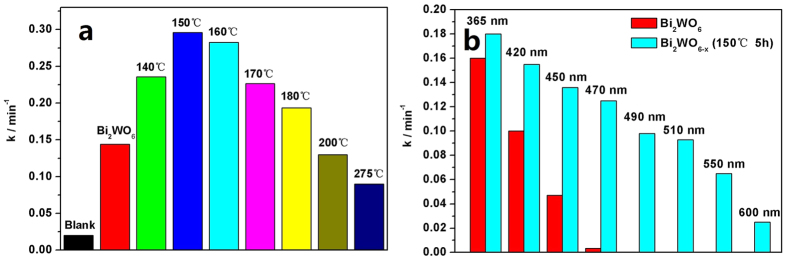
(**a**) The simulated solar photocatalytic activities of Bi_2_WO_6_ and Bi_2_WO_6−x_ samples after hydrogen reduction at various temperatures for 5 h on the degradation of 2, 4–DCP, under 500 W Xe lamp with an AM1.5 filter; (**b**) The photoactivities of Bi_2_WO_6_ and Bi_2_WO_6−x_ samples on the degradation of 2, 4–DCP, under different wavelength irradiation.

Fig. 2a shows the simulated sunlight photocatalytic performance of Bi_2_WO_6_ and Bi_2_WO_6−x_ samples on the degradation of 2, 4–DCP, utilizing 500 W Xe lamp an AM1.5 filter. It can be seen that the influence law of the temperature on the photocatalytic activity is the same as that under visible light. The photoactivities of Bi_2_WO_6−x_ samples firstly increase and then begin to decline with the increased of the hydrogen reduction temperature. However, the difference here is the apparent rate constant *k* of Bi_2_WO_6−x_ reduction at 150 °C is to 0.30 min^−1^ and it is about 2.1 times as high as that of pristine Bi_2_WO_6_, which is higher than that under visible light (1.6 times). This indicates that the UV photoactivity of Bi_2_WO_6−x_ is improved, which can also be testified via the increased UV photoactivity of Bi_2_WO_6−x_ on the degradation of 2, 4–DCP under λ = 365 nm light, as shown in Fig. 2b.

To further investigate the extending of visible photoresponse range of Bi_2_WO_6−x_ (150 °C, 5 h), the photocatalytic performances on the degradation of 2, 4–DCP of it and pristine Bi_2_WO_6_ were investigated, under 500 W Xe lamp with different wavelength filter (Fig. 2b). It can be found that the photoactivity of Bi_2_WO_6−x_ shows visibly higher than that of pristine Bi_2_WO_6_ under λ < 450 nm. And the longer of the incident light wavelength, the higher of the enhanced photoactivity of Bi_2_WO_6−x_ (150 °C, 5 h). Since the energy band gap of pristine Bi_2_WO_6_ is about 2.8 eV, it cannot be excited by the light with λ > 470 nm, thus it has hardly photocatalytic activity under 470 nm light irradiation. However, Bi_2_WO_6−x_ (150 °C, 5 h) photocatalyst shows distinctly photocatalytic activity (λ > 470 nm). Even under λ > 600 nm irradiation, it still exhibits observable photoactivity. In sum, the visible photoresponse range is extended from 450 nm for pristine Bi_2_WO_6_ to more than 600 nm for Bi_2_WO_6−x_ (150 °C, 5 h) induced by the introduction of surface oxygen vacancies.

### Formation and Structure of Surface Oxygen Vacancy

**Figure 3:**
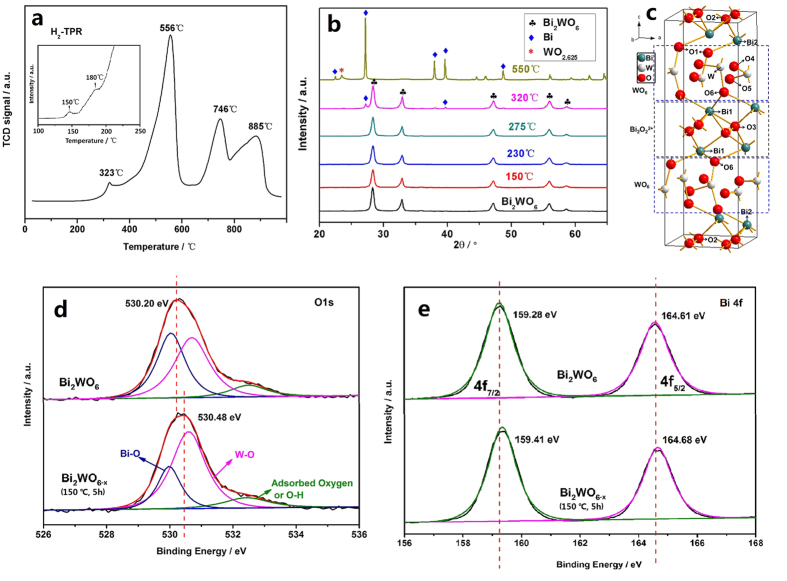
(**a**) H_2_–TPR profile of Bi_2_WO_6_ sample, the inset shows an enlarged TCD signal; (**b**) The XRD spectra of Bi_2_WO_6_ and Bi_2_WO_6−x_ samples; (**c**) The unit cell of Bi_2_WO_6_. Blue, white and red spheres represent Bi, W and O atoms; (**d**) O 1s XPS spectra of Bi_2_WO_6_ and Bi_2_WO_6−x_ (150 °C, 5 h) samples. The red curve is the fitting of experimental data for samples, which is decomposed into a superposition of three peaks shown as blue, pink and green curves; (**e**) Bi 4f XPS spectra of Bi_2_WO_6_ and Bi_2_WO_6−x_ (150 °C, 5 h) samples.

To investigate the hydrogen reduction process, temperature–programmed reduction (TPR) of the Bi_2_WO_6_ nanoplates was investigated (Fig. 3a). The H_2_–TPR profile of Bi_2_WO_6_ sample shows four reduction peaks, the main sharp peak is at about 556 °C, and the other three are at about 323, 746 and 885 °C, respectively. In addition, from the enlarged thermal conductivity detector (TCD) signal in the range of 100 ~ 250 °C (the inset of Fig. 3a), it can be found that two other little reduction peaks emerge at 150 °C and 180 °C, respectively. The six peaks are attributed to the partial loss of oxygen atoms or the reductive decomposition for the Bi_2_WO_6_ samples, which can be confirmed from the surface atomic concentration obtained from the X–ray photoelectron spectroscopy (XPS) (Table 1) and the X–ray diffraction (XRD) patterns of Bi_2_WO_6_ and Bi_2_WO_6−x_ samples (Fig. 3b). From Table 1, it can be seen that surface atomic concentration ratio of Bi4f:W4f:O1s changes from 1:0.384:3.07 for pristine Bi_2_WO_6_ to 1:0.385:2.90 for Bi_2_WO_6−x_. Compared with Bi_2_WO_6,_ the surface atomic concentration ratio of Bi4f:W4f for Bi_2_WO_6−x_ (150 °C, 5 h) has hardly any change, but that of Bi4f:O1s obviously increases, indicating that the surface oxygen atoms are removed. This confirms the formation of the surface oxygen vacancies. Fig. 3b shows no phase transformation or any impurity is observed for Bi_2_WO_6−x_ samples after hydrogen reduction at temperature range from 150 to 275 °C for 5 h, so here only oxygen atoms are removed from Bi_2_WO_6_, generating oxygen vacancies with different number and degrees. However, after 320 °C 5 h hydrogen reduction, a small amount of metal Bi is generated; even after 550 °C 5 h hydrogen reduction, the Bi–O bonds are ruptured totally and part of the W–O bond was also fractured, thus Bi_2_WO_6_ is reduced to the mixture of metal Bi and WO_2.625_. These phenomena are closely related with the structure of Bi_2_WO_6_ and the bond energy between two atoms.

From Fig. 3c, it can be visually seen that there are six nonequivalent O atoms in Bi_2_WO_6_, and they are connected with Bi1, Bi2 and W atoms, respectively. Based on the bond length of Bi–O or W–O (supporting information Table S1) and the bond angle of O–Bi–O (or O–W–O) (Table S2)^33^, O atoms should be firstly removed from Bi atoms, then partly leave from W atom, and the losing order is from surface O atoms to bulk O atoms inch by inch, thus generating oxygen vacancies with different number and degrees. This is echo with the results of the H_2_–TPR and XRD of as–prepared samples.

The change of surface chemical bonding of Bi_2_WO_6_ photocatalysts before and after hydrogen reduction was investigated with x-ray photoelectron spectroscopy (XPS). The O 1s and Bi 4f XPS spectra of Bi_2_WO_6−x_ (150 °C, 5 h) shift to a higher binding energy from 530.20 eV, 159.28 eV to 530.48 eV, 159.41 eV, respectively (Fig. 3d,e), which are contributed to the formation of neighboring oxygen vacancies with a high electron-attracting effect. The W 4f XPS spectra are almost identical Fig. S1, which indicates that W atom have a similar bonding environment after hydrogenation reduction. Furthermore, the O1s peak can be separated into three peaks at 530.05, 530.69 and 532.29 eV. The two lower binding energy centers at 530.05 eV, 530.69 eV belong to the coordination of oxygen in Bi–O and W–O, respectively^34^,^35^. The higher binding energy centered at 532.29 eV can be attributed to the coordination of oxygen in O–H or adsorbed oxygen^34^,^35^,^36^. It can be clearly found that the contribution of Bi–O is decreased for Bi_2_WO_6−x_ (150 °C, 5 h), which results from the removed of the O atom connecting with Bi atom. This phenomenon further confirms the formation of oxygen vacancies and reveals the source of oxygen vacancies.

**Figure 4:**
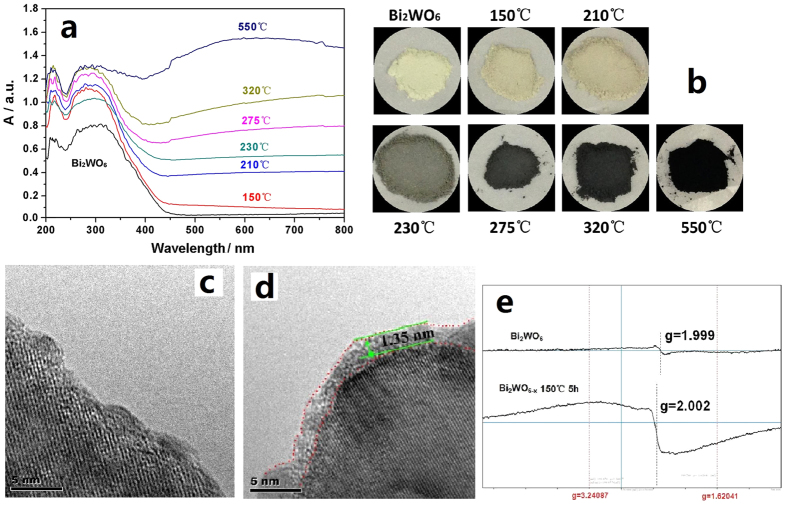
(**a**,**b**) UV–DRS and the photoes of Bi_2_WO_6_ and Bi_2_WO_6−x_ samples; (**c**,**d**) The HR–TEM of Bi_2_WO_6_ and Bi_2_WO_6−x_ (150 °C for 5 h); (**e**) *In situ* EPR spectra of Bi_2_WO_6_ and Bi_2_WO_6−x_ (150 °C for 5 h), at 77 K.

The UV–DRS of Bi_2_WO_6_ and Bi_2_WO_6−x_ powders after hydrogen reduction at different temperature were shown in Fig. 4a. The absorbance of as–prepared samples is gradually increased with the enhanced hydrogen reduction temperature. However, the photocatalytic activities are not wholly increase with the enhanced of the hydrogen reduction temperature. Therefore, the conclusion can be drawn that the increase of the absorbance cannot always result in the enhancement of the photoactivity. Here, the largely enhanced absorbance is mainly attributed to the samples color deepened from faint yellow pristine Bi_2_WO_6_ to gray–yellow Bi_2_WO_6−x_, finally turn into black powders composed by Bi and WO_2.625_ after hydrogen reduction at different temperature (Fig. 4b). This is because the electron–trapped oxygen vacancy can bind one or two electrons, giving rise to singly charged (F^+^ centers) or to neutral F^0^ center (known as a color center)^37^,^38^,^39^. Nevertheless, although the absorbance of Bi_2_WO_6−x_ (150 °C, 5 h) sample only shows a small improvement and the band edge also slightly red–shift, the photocatalytic performance are significantly boosted, which are caused by the generation of surface oxygen vacancies.

From Fig. 4c,d the HR–TEM of Bi_2_WO_6_ and Bi_2_WO_6−x_ (150 °C, 5 h) samples, it can be intuitively seen that pristine Bi_2_WO_6_ reveals perfect lattice features, however, the edge of Bi_2_WO_6−x_ (150 °C, 5 h) particles becomes disordered (thickness about 1.35 nm), which indicates that the surface structure of Bi_2_WO_6−x_ (150 °C, 5 h) is damaged and surface oxygen vacancies are formed. Electron paramagnetic resonance (EPR) is a sensitive and direct technique to monitor various behaviors to the presence oxygen defects^40^. To further confirm the existence of oxygen vacancy, *in situ* EPR of as–prepared samples was surveyed at 77 K in liquid N_2_, as shown in Fig. 4e. Up to now, *in situ* EPR signal of Bi_2_WO_6_ powders has rarely been reported. However, it is reported previously that the g factor ~2.001 is attributed to oxygen vacancy for TiO_2_ 41,42 and ZnO^43^,^44^. In this work, the strengthening and broadening of the EPR signal for Bi_2_WO_6−x_ (150 °C, 5 h) about g ~2.002 can also be attributed to the electron–trapped center at the site of oxygen vacancies, as reported previously by our group about oxygen–deficient BaAl_2_O_4_ 45.

To detect whether the Bi_2_WO_6−x_ (150 °C, 5 h) sample contains some hydrogen atoms after hydrogen reduction, the mass spectrum (MS) of hydrogen in the vent gas during the process of H_2_–TPD of Bi_2_WO_6−x_ (150 °C, 5 h) were performed. From supporting information Fig. S2, it can be seen that the hydrogen signal has hardly any change in the vent gas, implying that the hydrogen element content in Bi_2_WO_6−x_ (150 °C, 5 h) can be negligible. On the other hand, the hydrogen–related defects (O–H) also can be detected and quantitatively estimated by IR spectra. As is well known, the peaks around 3480 cm^−1^ and 1620 cm^−1^ are OH stretching vibration and bending vibration. However, from the IR spectra of as–prepared samples from 600 nm to 3600 nm (supporting information Fig. S3), it can not find the distinct signal of OH vibration peak. In addition, the 1385 cm^−1^ is also expected to be hydrogen–related defects modes, which were expected an additional band of OH vibration^46^,^47^,^48^. Compared with pristine Bi_2_WO_6_, the intensities of 1385 cm^−1^ peak signal (hydrogen–related defects) of Bi_2_WO_6−x_ (150, 230 °C) show hardly any change (still no signal), indicating that the number of hydrogen–related defects in Bi_2_WO_6−x_ (150 °C, 5 h) nanoplates with high photoactivity and photocurrent can be neglected (the inset of Fig. S3). Nevertheless, after hydrogen reduction at 275 °C for 5 h, the Bi_2_WO_6−x_ exhibits a little peak at 1385 cm^−1^, implying that it possesses a small number of hydrogen–related defects, which plays a supplementary role in the decrease of the photocatalytic activity^45^.

### Mechanism of Enhanced Photocatalytic Activity and Efficiency

The photocatalytic mechanism can be elucidated by the trapping experiments of radicals and holes. The main oxidative species in the photocatalytic process could be detected through the trapping experiments of hydroxyl radicals (•OH), holes and superoxide radical (•O_2_^−^) by using t–BuOH(•OH scavenger)^49^, HCOOH(hole scavenger)^50^ and purging N_2_ gas(•O_2_^−^ scavenger)^51^, respectively. Fig. S4a shows that the photoactivity of Bi_2_WO_6_ is greatly prevented by the addition of HCOOH, however, the addition of t–BuOH and purging of N_2_ gas only cause a small change in the photodegradation of 2, 4–DCP. The result suggests that the photogenerated holes are the main oxidative species of Bi_2_WO_6_ system. On the other hand, the photoactivity is also greatly inhibited by the addition of HCOOH in Bi_2_WO_6−x_ (150 °C, 5 h) system (Fig. S4b), so the main oxidative species is also holes, which is the same as that of in pristine Bi_2_WO_6_ system. •O_2_^−^ and •OH play the assistant role. Therefore, the photocatalytic degradation mechanism of Bi_2_WO_6−x_ (150 °C, 5 h) on 2, 4–DCP is not changed and the main oxidative species is still holes.

As discussed above, the degradation mechanism of Bi_2_WO_6−x_ (150 °C, 5 h) is the same as that of pristine Bi_2_WO_6_, and the particle size, the crystal phase structure (from the XRD spectra, as shown in Fig. 3b) are not distinctly changed. The small enhanced adsorption ability for 2, 4–DCP from 4% of Bi_2_WO_6_ to 10% of Bi_2_WO_6−x_ (150 °C, 5 h) as shown in Fig. S5 is resulted from the slightly enlarged of the surface area (Bi_2_WO_6_: 21.53 m^2^/g; Bi_2_WO_6−x_ (150 °C, 5 h): 23.45 m^2^/g) and the decrease of the zeta potential (Bi_2_WO_6_: 0.42 mV; Bi_2_WO_6−x_ (150 °C, 5 h): –12.80 mV) (as shown in Table 2) due to the formation of surface oxygen vacancies, which is in favor of the enhanced photoactivity.

**Figure 5:**
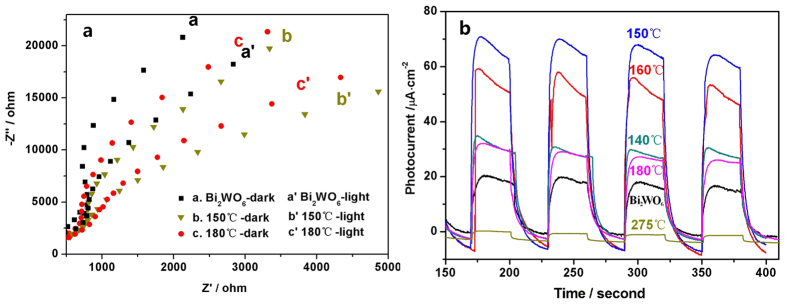
(**a**) EIS of pristine Bi_2_WO_6_ and Bi_2_WO_6−x_ (150 and 180 °C, 5 h) after deposition on ITO electrodes, with and without visible light irradiation (λ > 420 nm); (**b**) Photocurrents of Bi_2_WO_6_ and Bi_2_WO_6−x_ samples after deposition on ITO electrodes, under visible light (λ > 420 nm).

The separation efficiency of photogenerated electron and hole pairs also plays an important role in the enhancement of photocatalytic activity, which can be investigated by the typical electrochemical impedance spectroscopy (EIS). Fig. 5a shows the EIS responses of pristine Bi_2_WO_6_ and Bi_2_WO_6−x_ electrodes with and without visible irradiation. In each case, there is one arc on the EIS plane, indicating that the surface charge transfer is the rate–determining step in the photocatalytic reaction. A necessary step for semiconductor photocatalytic performance is the generation and separation of photogenerated electron–hole pairs. The arc radiuses of the Bi_2_WO_6−x_ (150 and 180 °C) electrode both are smaller than that of the pristine Bi_2_WO_6_ electrode regardless of whether with or without visible-light irradiation, and the order is Bi_2_WO_6−x_ (150 °C) < Bi_2_WO_6−x_ (180 °C) < pristine Bi_2_WO_6_. The smaller the arc radius of an EIS Nyquist plot, the higher the efficiency of charge separation^52^,^53^. Thus, in the case of Bi_2_WO_6−x_, the photogenerated electron–hole pairs are easier separated and transferred to the samples surface, and the photoactivities both are higher than that of pristine Bi_2_WO_6_. Therefore, the enhanced photocatalytic performance is mainly attributed to the increase of the charge separation efficiency due to the broadening of the valence band (VB) width induced by surface oxygen–vacancy states, which will be discussed in the following part.

Furthermore, the photocurrent also can be considered as an indicator of the recombination rate of photogenerated electron–hole pairs^37^. Under visible light, **t**he photocurrent densities of Bi_2_WO_6−x_ (hydrogen reduction at 140, 150, 160, 180 °C, respectively) electrodes are all higher at different level than that of pristine Bi_2_WO_6_ electrode (Fig. 5b). Especially, Bi_2_WO_6−x_ (reduction at 150 °C) shows the highest photocurrent, which is about 3.5 times as high as that of pristine Bi_2_WO_6_. However, Bi_2_WO_6−x_ (reduction at 275 °C) electrode shows slightly lower photocurrent than that of pristine Bi_2_WO_6_ electrode. The enhanced or reduced photocurrents demonstrate that the increase or decrease of the photogenerated carriers transport rate, which are associated with the number and kind of oxygen vacancies. These results are well consistent with the change (improve or reduce) of the photocatalytic activities for the as–prepared samples (Fig. 1a) and the EIS plot (Fig. 5a).

**Figure 6:**
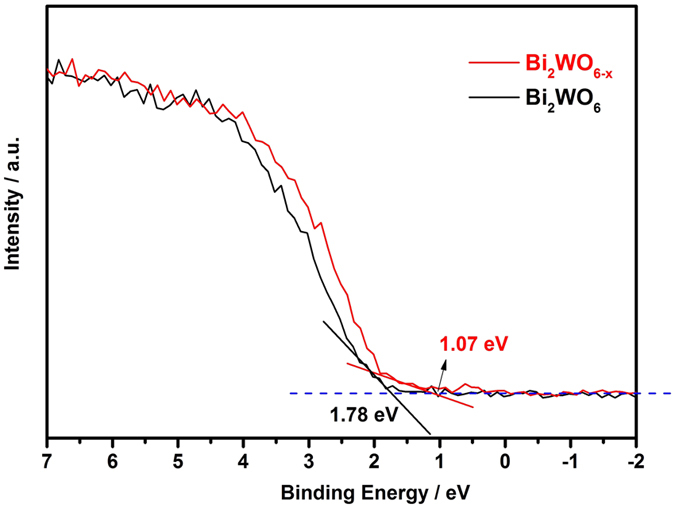
Valence-band XPS spectra of the pristine Bi_2_WO_6_ and Bi_2_WO_6−x_ (150 °C for 5 h).

The density of states (DOS) of the valence band of Bi_2_WO_6_ photocatalysts were measured by the valence band XPS. The Bi_2_WO_6_ displays valence band DOS with the edge of the maximum energy at about 1.78 eV, however, the edge of the valence band energy for Bi_2_WO_6−x_ (150 °C, 5 h) shifts toward the vacuum level at approximately 1.07 eV (Fig. 6), indicating the valence band maximum rise with low density of states.

**Figure 7:**
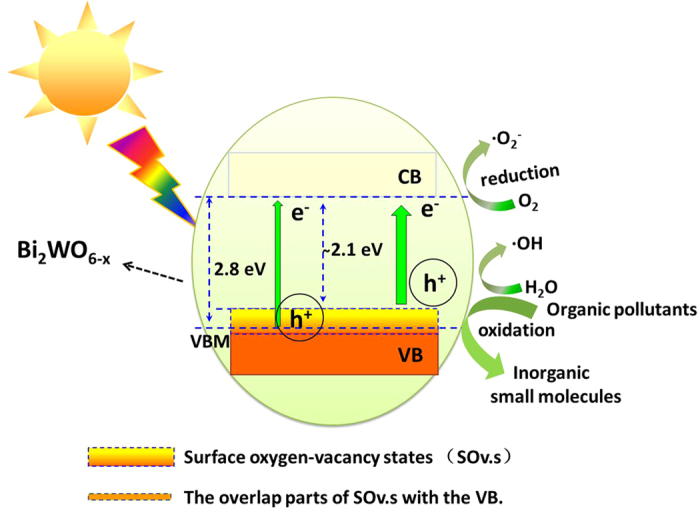
Schematic diagram illustrating the mechanism of charge separation and photocatalytic reaction of Bi_2_WO_6−x_ photocatalyst, under sun light irradiation.

Based on the above discussions, the proposed schematic diagram for the mechanism of the enhanced photocatalytic activity and the efficiency of Bi_2_WO_6−x_ photocatalyst are provided in Fig. 7. Surface oxygen vacancy is a shallow defect, which may be below the conduction band minimum (CBM) or above the valence band maximum (VBM). It was reported previously by Jung and coauthor that the rising of VBM and the reduced of band gap over anatase TiO_2_ from O-terminated surface to Ti-termination surface has been observed image by the scanning tunneling microscopy^54^. The band gap of LiTi_2_(PO_4_)_3_ significantly reduced due to the formation of oxygen–vacancy states above the valence band, under poor oxygen conditions^55^. And our group also has been found that the surface oxygen-defects states will be generated on the VBM for ZnO and BiPO_4_ through experimental and theoretical calculation^28^,^29^,^30^,^56^. Furthermore, the work shows that the photoresponse wavelength range is extended as well as the photoactivity is enhanced for Bi_2_WO_6−x_ photocatalyst. The extending of the photoresponse wavelength range (from 450 nm to about 600 nm) can be attributed to the shallow surface oxygen–vacancy states above and partly overlapping with the VB of Bi_2_WO_6_, resulting in the rise of the VBM. Simultaneously, the rising of the VBM also can results in the expanding the VB width. Therefore, the transport rate of photogenerated carriers improved, leading to the enhancement of separation efficiency of photogenerated electron–hole pairs, thus the photocatalytic performance also is improved.

In summary, the visible photoresponse range was extended and the photoactivity was also enhanced for Bi_2_WO_6_ nanoplates via surface oxygen vacancies. The visible-light wavelength range is expanded from 450 nm for pristine Bi_2_WO_6_ to 600 nm for Bi_2_WO_6−x_. The photoactivity of Bi_2_WO_6−x_ (150 °C, 5 h) is 2.1 times as high as that of pristine Bi_2_WO_6_. The expanded the visible-light range and the enhanced photoactivity both are resulted from the production of the surface oxygen vacancies. These are because surface oxygen–defect states are located above and partly overlapping with the VB, resulting in the rising of the valence band maximum with low density of states and the broadening of the VB. Furthermore, the generation of oxygen vacancies also can slightly increase the adsorbance on 2, 4–DCP, which is a supplement to the enhanced of the photoactivity.

## Methods

### Preparation of photocatalysts

Square Bi_2_WO_6_ nanoplates (surface area 21.53 m^2^/g) were synthesized by the simple hydrothermal method^14^. Bi_2_WO_6_ with oxygen vacancy samples were prepared as follows: (1) the temperature programmed reduction (TPR) measurement (ChemiSorb 2720, Micromeritics, America) using hydrogen gas was performed in a specially designed quartz tube with 0.073 g of Bi_2_WO_6_. (2) Based on the H_2_–TPR graph, hydrogen reduction process was performed to prepare oxygen–vacancy Bi_2_WO_6_ samples. Firstly, the synthesized Bi_2_WO_6_ samples were pretreated by He gas at 120 °C for 2 h. Then H_2_/Ar mixture gas was introduced and the temperature from 25 ± 0. 5 °C to designed temperature at a rising rate of 10 °C · min^−1^, the time was kept for 1 ~ 9 h at 2 h intervals. Finally, the samples were cooled down to room temperature naturally, maintaining H_2_/Ar mixture gas.

### Characterization

Ultraviolet–visible diffuse reflectance spectroscopy (UV–DRS) was performed on Hitachi U–3010, BaSO_4_ was used as reference. The crystallinity and purity of the as-prepared samples was characterized by X-ray diffraction (XRD) on Bruker D8-advance diffractometer using Cu Ka radiation (λ = 1.5418 Å). X-Ray photoelectron spectroscopy (XPS) was obtained using a Quantera (ULVAC-PHI, Japan). An Al Ka X-ray source with a power of 25 W was used. The pass energy of the analyzer was set at 37.25 eV and the base pressure of the analysis chamber was better than 4 × 10^–8^ Torr. The binding energies were referenced to the C1s line at 284.8 eV from adventitious carbon. For our XPS data, all curves were defined as 20% Lorentzian, 80% Gaussian. The curve fitting was done using XPSpeak software, with Newton’s iteration method and 300 iterations. A high–resolution transmission electron microscope (HR–TEM, JEM 2010F) were operated at an accelerating voltage of 200 kV. *In situ* electron paramagnetic resonance (EPR) measurement was carried out using an endor spectrometer (JEOL ES–ED3X) at 77 K in liquid nitrogen. The g factor was obtained by taking the signal of manganese as standard. Atmospheric gas analysis system (HIDEN QIC20–MS) was utilized to survey the hydrogen in the vent gas formed in the process of H_2_-TPD of Bi_2_WO_6−x_ (150 °C for 5 h). The photocurrents and electrochemical impedance spectroscopy (EIS) were measured on an electrochemical system (CHI–660B, China). Surface area was determined with the Brunauer–Emmett–Teller (BET) method, porosity measurements (TriStar II3020, Micromeritics). The zeta potentials were measured by the monitor particle method using an electrophoretic light scattering spectrophotometer (Size–100, Horiba).

### Catalytic Evaluation

The photocatalytic performances of as–prepared samples were evaluated by the decomposition of 2, 4–DCP, in solution under visible light and simulated solar irradiation. Visible light source was obtained from a 500 W Xe lamp with a 420 nm cutoff filter. Simulated solar irradiation was obtained from the Xe lamp with an AM1.5 filter. 25 mg photocatalyst was added into prepared 50 mL 15 ppm of 2, 4–DCP aqueous solution. Before the light irradiation, the suspensions were firstly ultrasonic dispersed in dark for 10 min, then magnetically stirred for 15 min to reach the adsorption–desorption equilibrium. At given time intervals, 3 mL aliquots were sampled and centrifuged to remove the photocatalyst particles. Synchronously, the filtrated 2, 4–DCP solutions were analyzed by the HPLC (Lumtech) analysis with a UV detector at 284 nm. The Venusil XBP–C18 reversed phase column was used. The mobile phase was methanol and water (75:25, v/v) with a flow rate of 1.0 mL · min^−1^.

Photoelectrochemical measurements were carried out in a conventional three–electrode system. ITO/Bi_2_WO_6_ or ITO/Bi_2_WO_6−x_ electrodes served as the working electrode. A platinum wire as counter electrode and a standard calomel electrode (SCE) as reference electrode were utilized in the photoelectric studies. 0.1 M Na_2_SO_4_ was used as the electrolyte solution. ITO/Bi_2_WO_6_ or ITO/Bi_2_WO_6−x_ electrodes were prepared by a dip–coating method: 5 mg of photocatalyst was suspended in 1 mL deionized water to make slurry, and then the slurry was dip–coated onto a 2 cm × 4 cm ITO glass electrode. The as–prepared electrodes were naturally dried and subsequently calcined at 80 °C for 5 h in vacuum drying oven. All investigated electrodes were of similar thickness (0.8–1 μm).

## Additional Information

**How to cite this article**: Lv, Y. *et al*. Fabrication of Wide-Range-Visible Photocatalyst Bi_2_WO_6-x_ nanoplates via Surface Oxygen Vacancies. *Sci. Rep.* **6**, 19347; doi: 10.1038/srep19347 (2016).

## Supplementary Material

Supplementary Information

## Figures and Tables

**Table 1:** Surface atomic concentration of Bi_2_WO_6_ and Bi_2_WO_6−x_ (150 °C, 5 h) come from XPS.

Sample	Bi4f	W4f	O1s	Bi4f:W4f:O1s
Bi_2_WO_6_	16.53	6.35	50.87	1:0.384:3.07
Bi_2_WO_6−x_ (150 °C, 5 h)	16.65	6.42	48.29	1:0.385:2.90

**Table 2:** The comparison of BET and Zeta potential for Bi_2_WO_6_ and Bi_2_WO_6−x_ samples.

Sample	BET (m^2^/g)	Zeta potential (mV)
Bi_2_WO_6_	21.53	0.42
Bi_2_WO_6−x_ (150 °C, 5 h)	23.54	−12.80
