# Supplementary material for: Fabrication of Wide–Range–Visible Photocatalyst Bi2WO6−x nanoplates via Surface Oxygen Vacancies
Source: Sci Rep. 2016 Jan 18;6:19347. doi: 10.1038/srep19347 (PMC4726091; doi:10.1038/srep19347)
Supplement: Supplementary Information [file srep19347-s1.doc]

**Fabrication of Wide–Range–Visible Photocatalyst Bi_2_WO_6-x_ nanoplates via Surface Oxygen Vacancies**

Yanhui Lv^1,2^, Wenqing Yao^1^, Ruilong Zong^1^, and Yongfa Zhu^1^*

*^1^Department of Chemistry, Beijing Key Laboratory for Analytical Methods and Instrumentation, Tsinghua University, Beijing, 100084, People’s Republic of China.*

*^2^Key Laboratory of Photochemistry Beijing National Laboratory for Molecular Sciences Institute of Chemistry, Chinese Academy of Sciences, Beijing 100190, People’s Republic of China.*

** E-mail:* [*zhuyf@tsinghua.edu.cn*](mailto:zhuyf@tsinghua.edu.cn) *Tel: +86 010 62787601 Fax: +86 010 62787601*

[**Supporting**](javascript:void(0);) [**Information**](javascript:void(0);)


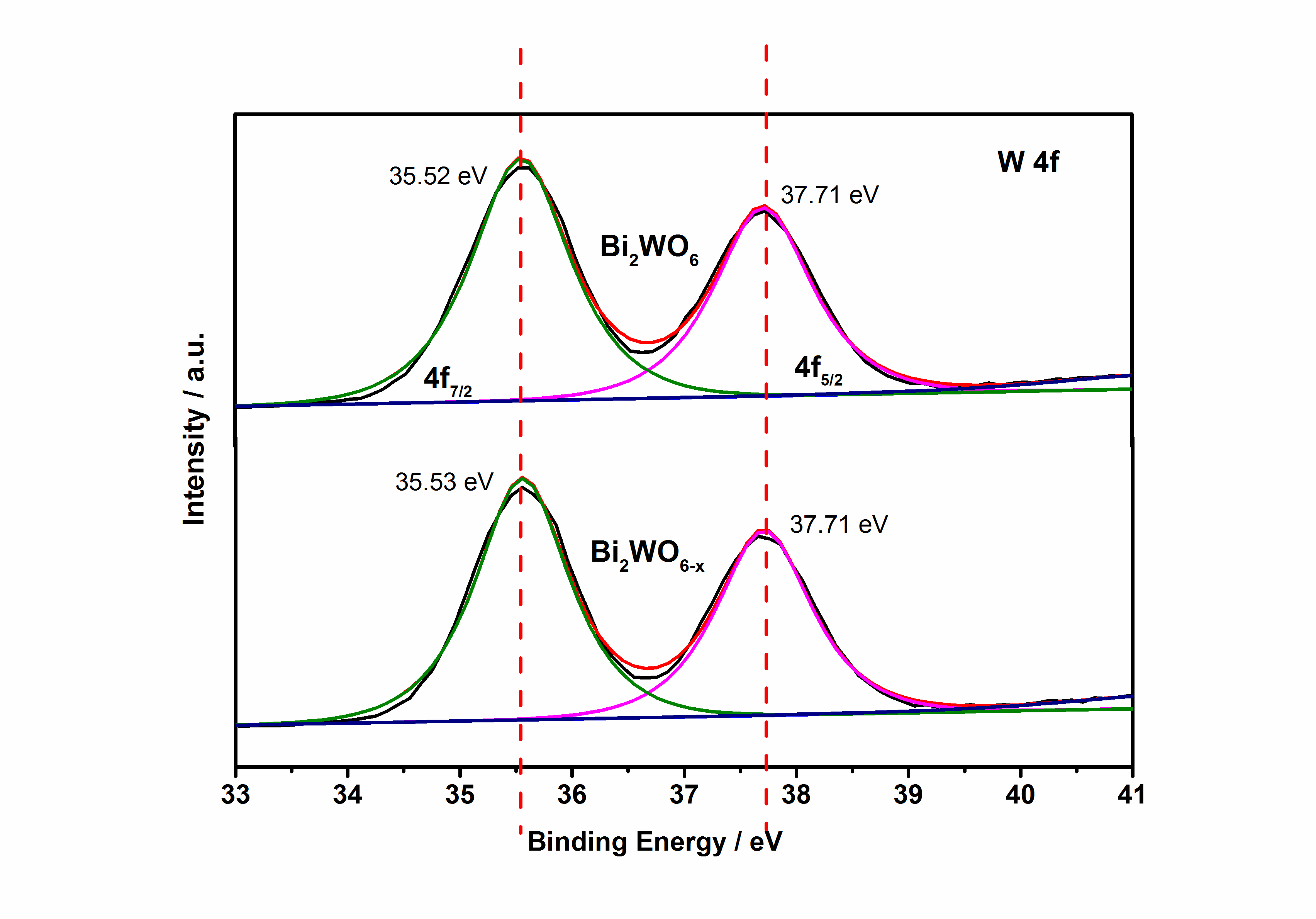


**Figure S1** W 4f XPS spectra of Bi_2_WO_6_ and Bi_2_WO_6-x_ samples.


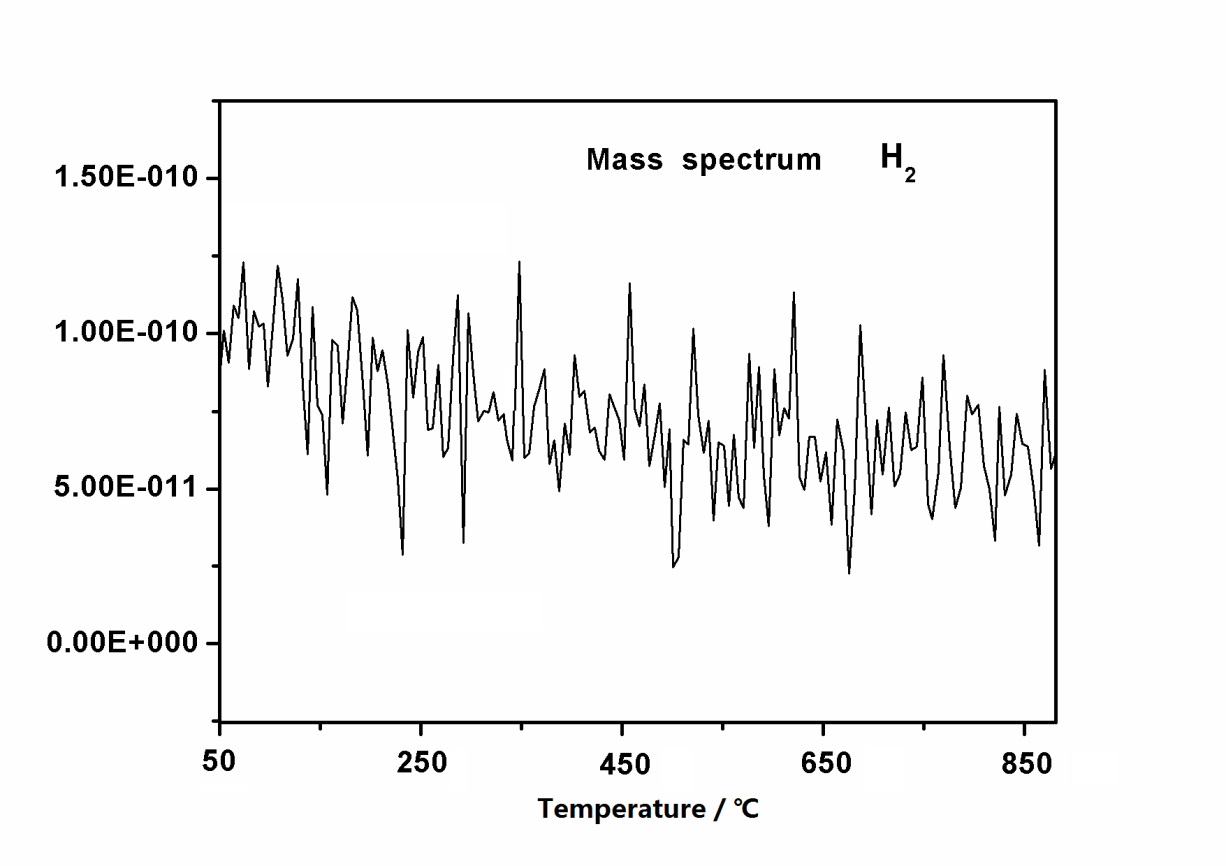


**Figure S2** The mass spectrum (MS) of H_2_ in the vent gas during the process of H_2_-TPD of Bi_2_WO_6-x_ (150 ºC for 5 h)

Furthermore, to detect whether the Bi_2_WO_6-x_ (150 ºC, 5 h) sample contains hydrogen (H) after hydrogen reduction, the mass spectrum (MS) of hydrogen in the vent gas during the process of H_2_-TPD of Bi_2_WO_6-x_ (150 ºC, 5 h) were performed. From **Fig. S2**, it can be seen that the hydrogen mass has hardly any change in the vent gas, implying that the H element content in Bi_2_WO_6-x_ (150 ºC, 5 h) can be negligible.


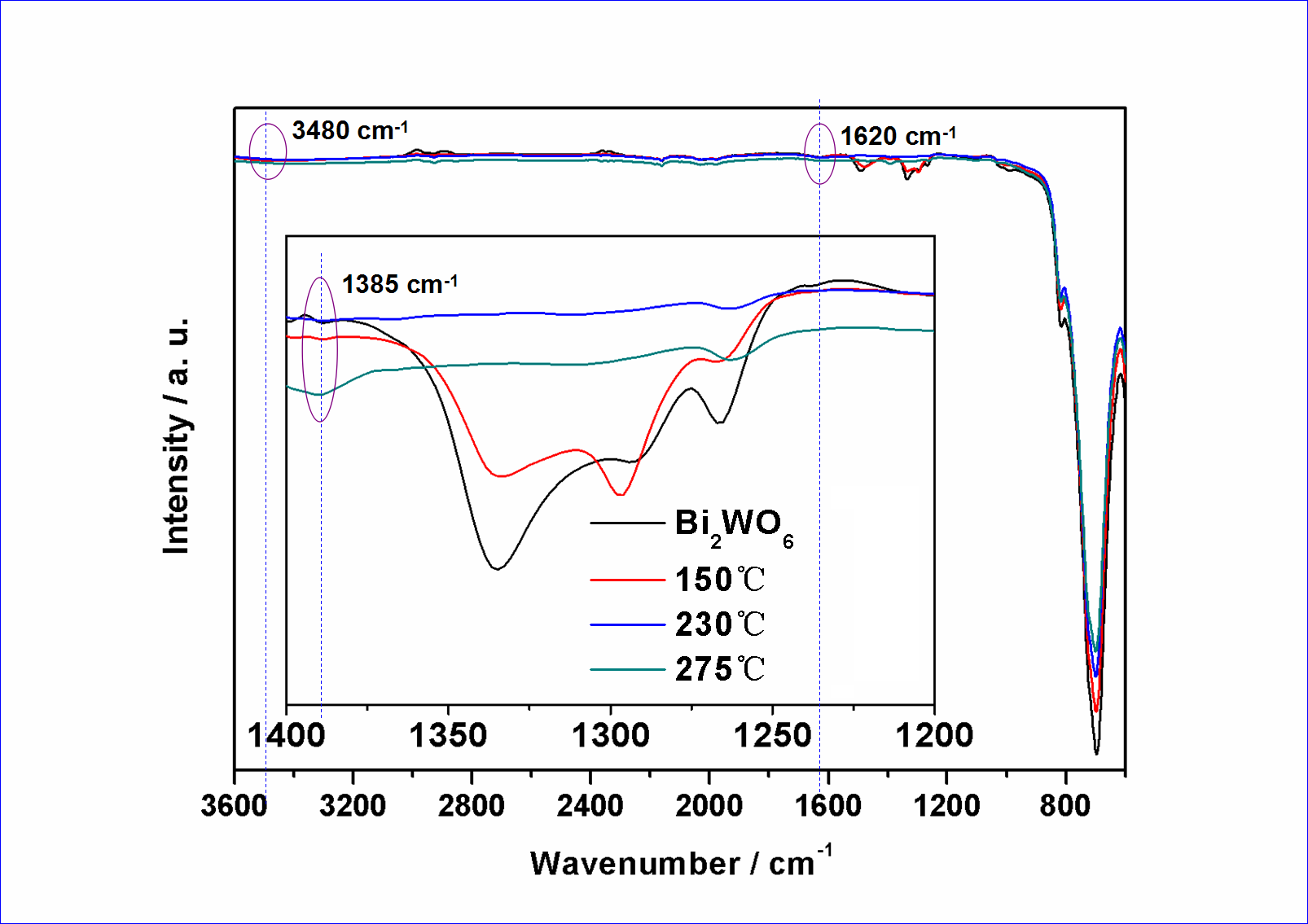


**Figure S3** The IR spectra of Bi_2_WO_6_ and Bi_2_WO_6-x_ samples, the wavenumber from 3600 to 600 cm^-1^; the inset shows the enlarged signal from 1400 to1200 cm^-1^.

The hydrogen-related defects (O-H) also can be detected and quantitatively estimated by IR spectrum. As is well known, the peaks around 3480 cm^−1^and 1620 cm^−1^ are OH stretching vibration and bending vibration. However, from the IR spectra of as-prepared samples from 600 nm to 3600 nm (**Fig. S3**), it can not find the visible signal of OH vibration peak. In addition, the 1385 cm^−1^ was also expected hydrogen-related defects modes, which are expected an additional band of OH vibration.^36-38^ Compared with pristine Bi_2_WO_6_, the intensities of hydrogen-related defects of Bi_2_WO_6-x_ (150, 230 ºC) show hardly any change (still no signal), indicating that the number of hydrogen-related defects in Bi_2_WO_6-x_ (150 ºC, 5 h) nanoplates with high photoactivity and photocurretn can be neglected (the inset of **Fig. S3**). Nevertheless, after hydrogen reduction treated at 275 ºC for 5 h, the Bi_2_WO_6-x_ exhibits a little peak at 1385 cm^-1^, implying that it possesses a small number of hydrogen-related defects, which plays a supplementary role in the decrease of the photocatalytic activity.^40^

**
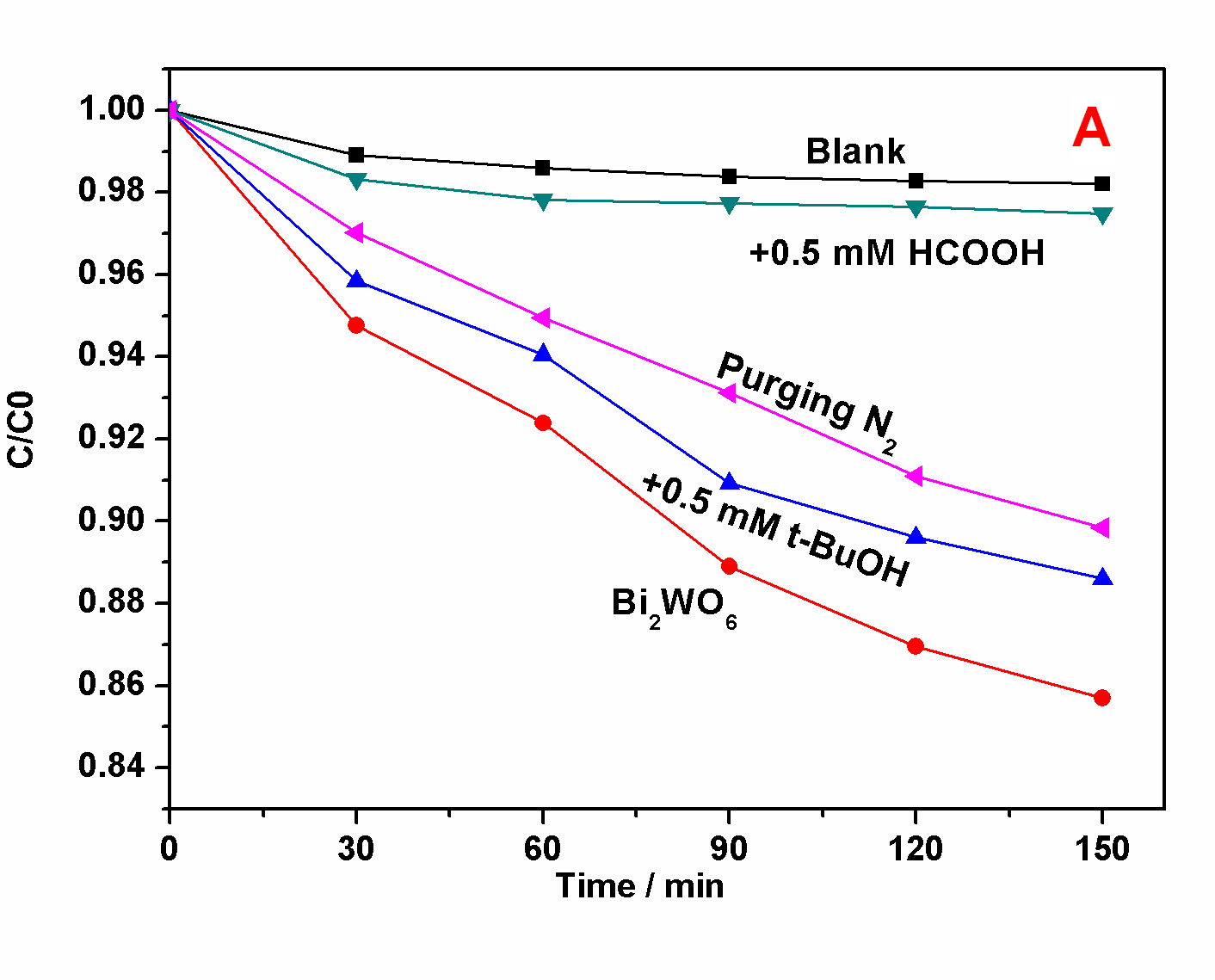

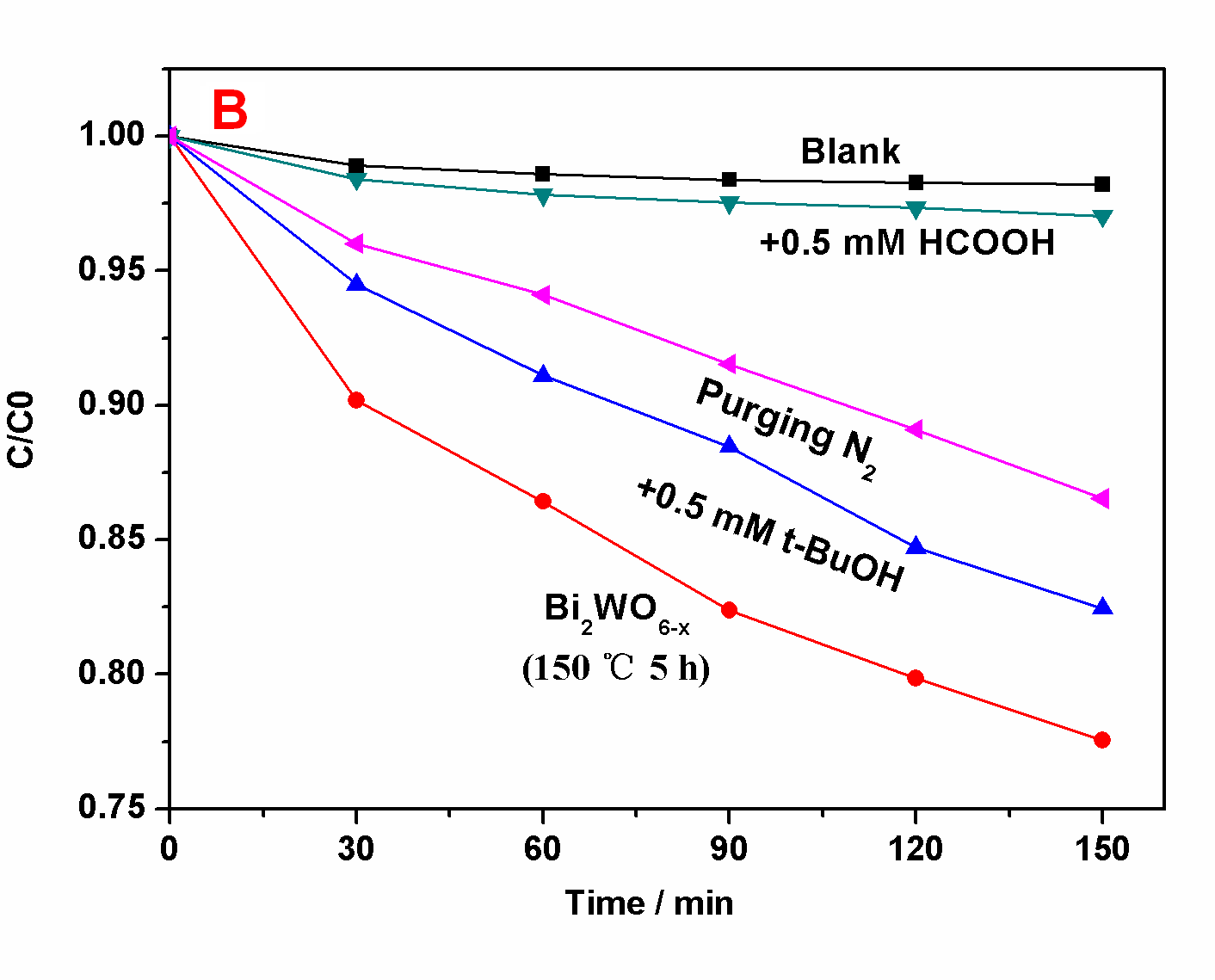
**

**Figure S4** Photogenerated carriers trapping in the system of photodegradation of 2, 4–DCP by **(a)** Bi_2_WO_6_ and **(b)** Bi_2_WO_6-x_ (150 ºC, 5 h), under visible light (λ > 420 nm).

The photocatalytic mechanism can be elucidated by trapping experiments of radicals and holes. The main oxidative species in the photocatalytic process could be detected through the trapping experiments of hydroxyl radicals (•OH), holes and [superoxide](javascript:void(0);) [radical](javascript:void(0);) (•O_2_^-^) by using t−BuOH (•OH scavenger), ^[39]^ HCOOH (hole scavenger) ^40^ and purging N_2_ gas (•O_2_^-^ scavenger),^41^ respectively. **Fig. S4a** showed that the photoactivity of Bi_2_WO_6_ could be greatly prevented by the addition of HCOOH, however, the addition of t–BuOH and purging of N_2_ gas only cause a small change in the photodegradation of 2, 4–DCP. The result suggests that the photogenerated holes are the main oxidative species of Bi_2_WO_6_ system. On the other hand, in Bi_2_WO_6-x_ (150 ºC, 5 h) system (**Fig. S4b**), the photoactivity is also greatly inhibited by the addition of HCOOH, so the main oxidative species is also holes, which is the same as that of in pristine Bi_2_WO_6_ system. •O_2_^-^ and •OH play the assistant role. Therefore, the photocatalytic degradation mechanism of Bi_2_WO_6-x_ (150 ºC, 5 h) on 2, 4–DCP is not changed and the main oxidative species is still holes.

**
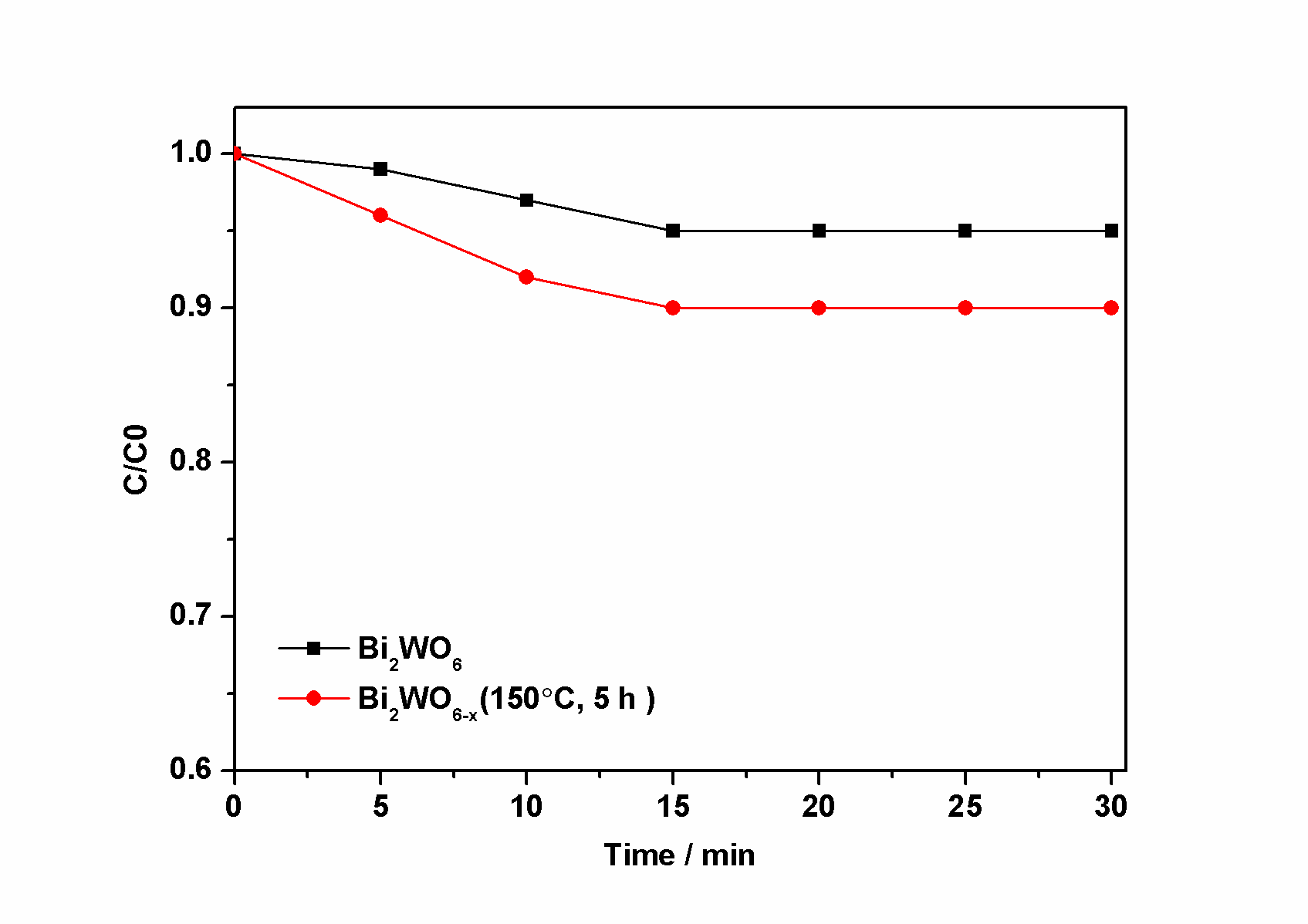
**

**Figure S5** The adsorption abilities of Bi_2_WO_6_ and Bi_2_WO_6-x_ (150 ºC, 5 h) samples on the degradation of 2,4-DCP solution, in dark.

The small enhanced adsorbability on 2, 4-DCP from 4 % of Bi_2_WO_6_ to 10 % of Bi_2_WO_6-x_ (150 ºC, 5 h) is resulted from the enlarge of the BET (Bi_2_WO_6_: 21.53 m^2^/g; Bi_2_WO_6-x_ (150 ºC, 5 h): 23.45 m^2^/g) and the decrease of the zata potential (Bi_2_WO_6_: 0.42 m^2^/g; Bi_2_WO_6-x_ (150 ºC, 5 h): -12.80 m^2^/g) due to the formation of surface oxygen vacancies, which is a bit beneficial to the improvement of the photoactivity.

**Table S1. Bond distance of atom-atom for Bi_2_WO_6_;**

| **Bond** [**distance**](app:ds:distance) **/ Å** | | | | |
| --- | --- | --- | --- | --- |
| **O1-Bi2**  **O1-W** | 2.47059  1.889 |  |  |  |
| **O2-Bi2** | 2.2232 | 2.2366 | 2.3695 | 2.4911 |
| **O3-Bi1** | 2.1402 | 2.2497 | 2.3363 |  |
| **O4-W** | 1.8086 | 2.1713 |  |  |
| **O5-W** | 1.7869 | 2.1469 |  |  |
| **O6-W** | 1.8522 |  |  |  |

**Table S2 Bond angle of atom-atom-atom for Bi_2_WO_6._**

|  | **Bond angle / °** | | | | |
| --- | --- | --- | --- | --- | --- |
| **O1-Bi2-O2** | 144.709 | 141.078 | 77.974 | 72.843 |  |
| **O2-Bi2-O2** | 113.287 | 111.287 | 75.511 | 73.354 | 68.310 |
| **O3-Bi1-O3** | 112.096 | 76.910 | 75.524 |  |  |
| **O4-W-O1** | 93.882 | 78.185 |  |  |  |
| **O4-W-O4** | 87.787 |  |  |  |  |
| **O4-W-O5** | 169.650 | 168.561 | 101.402 | 81.570 |  |
| **O4-W-O6** | 100.502 | 82.264 |  |  |  |
| **O5-W-O1** | 96.273 | 79.895 |  |  |  |
| **O5-W-O5** | 88.887 |  |  |  |  |
| **O5-W-O6** | 100.501 | 82.286 |  |  |  |
| **O6-W-O1** | 155.197 |  |  |  |  |

Bi_2_WO_6_ has six inequivalent O atoms, they are connected with Bi1, Bi2 and W atoms, respectively. The longer of the bond distance and the bigger of the bond angle, the weaker of the bond energy. Based on the bond length of O-Bi or O-W (**Table S1**) and the bond angle of O-Bi (or W)-O (**Table S2**), O atoms should be firstly removed from Bi atom, then partly leave from the W atom, and the losing order is from surface O to bulk O atoms inch by inch, thus generating oxygen vacancies with different number and degrees.
